# Successful Selective Embolization for Recurrent Hemarthrosis after Knee Arthroplasty

**DOI:** 10.1155/2019/8374709

**Published:** 2019-12-05

**Authors:** Cristian Barrientos, Maximiliano Barahona, Tomas Cermenati, Rodrigo Wulf, Jaime Hinzpeter

**Affiliations:** ^1^Orthopaedic Department at Hospital Clinico Universidad de Chile, Santos Dumont, 999 Santiago, Chile; ^2^Interventional Radiology Department at Hospital Clinico Universidad de Chile, Santos Dumont, 999 Santiago, Chile

## Abstract

Knee replacement has demonstrated to be a cost-effective treatment for severe knee osteoarthritis. Nevertheless, perioperative complications may occur, including recurrent hemarthrosis reaching an incidence between 0.3 and 1.6%. Success rate after conservative treatment has been reported to be above 80%, but in case of recurrence, computed tomography angiography, magnetic resonance angiography, and Doppler ultrasound have been used to conduct the diagnosis. Arthroscopy or selective embolization is used for treatment depending on the etiology of the bleeding. Open surgery is performed in the rare cases of failure of the above alternatives. The patient consulted seven months after total knee arthroplasty with sudden pain in the medial side of the knee. Infection was ruled out, and arthrocentesis shows hemarthrosis. Successful selective embolization of medial superior and lateral superior genicular artery was performed. After two years, the patients report 92 points in the Forgotten joint score, 0 in Womac pain, 1 in Womac stiffness, and 3 in Womac functional score.

## 1. Introduction

Knee replacement has demonstrated good functional results, increase in quality of life, and cost-effectiveness in treatment for severe knee arthrosis [1]. Nevertheless, perioperative complications may occur, including thromboembolism, infection, and vascular injury, which can compromise the clinical outcome and survival of knee arthroplasty. Repetitive hemarthrosis after knee arthroplasty is a frequent clinical presentation of vascular complications, reaching an incidence of 0.3-1.6% [2], and may trigger pain, joint stiffness, functional deterioration, or, although infrequently, infection [3].

The causes can be divided into general or local. General causes are related to genetics and systemic or acquired conditions, so it is crucial to investigate the current use of anticoagulant drugs and check the coagulation blood test status and history of blood dyscrasia. In order to establish the etiological diagnosis of local causes, the moment of presentation is essential. The early presentation includes direct vascular injury, pseudoaneurysm, and arteriovenous fistulae. The late presentation includes synovial impingement and mechanical causes [4].

Our purpose is to report a case of recurrent hemarthrosis after knee replacement, due to arterial-arterial fistulae in the medial and lateral genicular arteries, successfully treated with embolization.

## 2. Case

**Figure 1:**
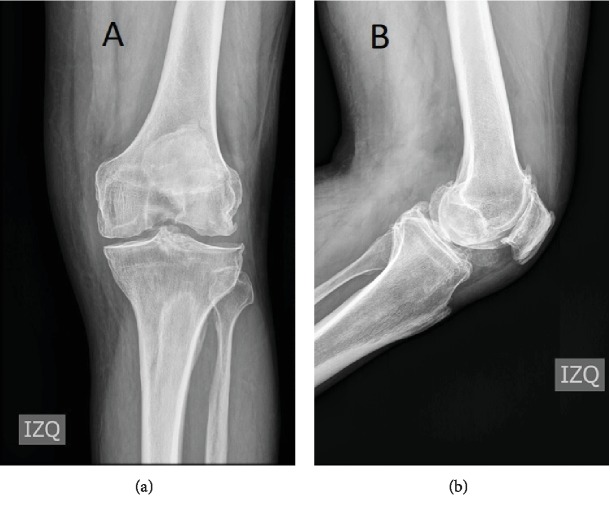
Preoperative radiographs show severe tricompartmental left knee osteoarthritis: (a) anteroposterior left knee view and (b) lateral left knee view.

A sixty-three-year-old man complains of atraumatic left knee pain for three years, which got worse in the last three months. Before surgery, the knee range of motion was -5° of extension and 100° of flexion. Preoperative radiographs show severe knee osteoarthritis (Figure 1). The patient was not under anticoagulation before surgery, and his hemoglobin was 13.1 gr/dl.

**Figure 2:**
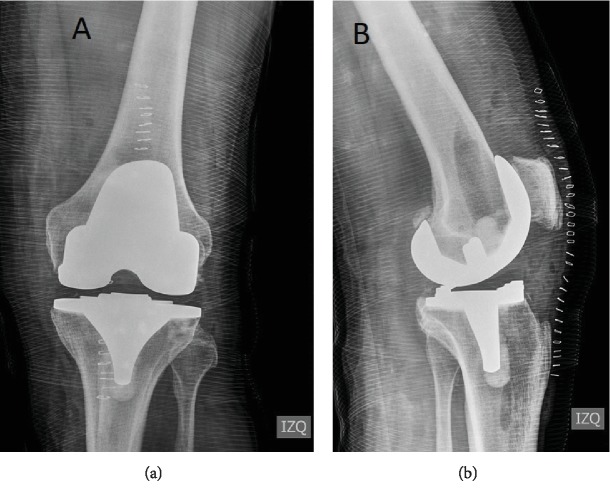
Immediately post knee arthroplasty radiograph: (a) anteroposterior left knee view and (b) lateral left knee view.

He underwent total knee arthroplasty (TKA), an anterior-stabilized (AS) bearing prosthesis was used (Vanguard®, Zimmer®). No acute complication was noted, and postsurgery radiographs are shown in Figure 2. At hospital discharge, nonsteroidal anti-inflammatory drugs and tramadol were used for pain relief, and 5000 IU daily of dalteparin for 21 days was indicated for thromboembolism prophylaxis. Three months after surgery, the patient was walking with no assistance and range of motion was 0° of extension and 130° of flexion. He complains of a mild nonpermanent pain in the proximal-medial side of the tibia, but he was able to resume work.

At seven-month postsurgery, he consulted in the emergency room due to an intense pain for three days in the proximal-medial side of the tibia. At the physical exam, the patient has no fever, the knee range of motion was 0°-100°, mild knee effusion was observed, and pain in the proximal-medial side of the tibia was found.

**Figure 3:**
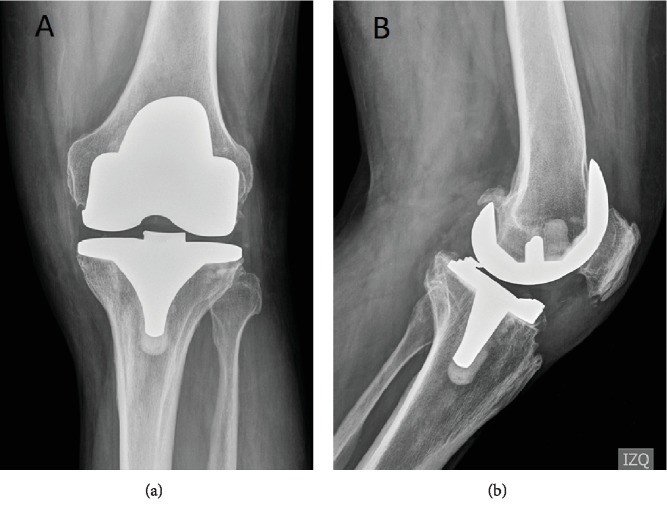
Radiograph seven months after left knee arthroplasty: (a) anteroposterior left knee view and (b) lateral left knee view. No signs of loosening or osteolysis were found.

Radiographs showed no signs of loosening or osteolysis (Figure 3). C-reactive protein (CPR) was 8.7 mg/l (normal value below 10 mg/l), ERS was 19 mm/hr, and hemoglobin was 12.2 gr/dl. An arthrocentesis to rule out infection was carried out, and hemarthrosis was found. The fluid analysis was 1800 white cells, and after 14 days, the cultures were negatives. Computed tomography was done; no signs of osteolysis, fracture, or prosthesis component maltorsion were found. The patient was readmitted; an angiography computed tomography (Angio-CT) was performed, but no arteriovenous malformation or active bleeding was found; nevertheless, hemarthrosis was found.

It was decided to perform a diagnostic arthroscopy. Two anterior and two posterior portals were used, but no active bleeding was found, only clots. The synovial tissue had typical aspects, but two samples were taken for biopsies. The patient was discharged.

**Figure 4:**
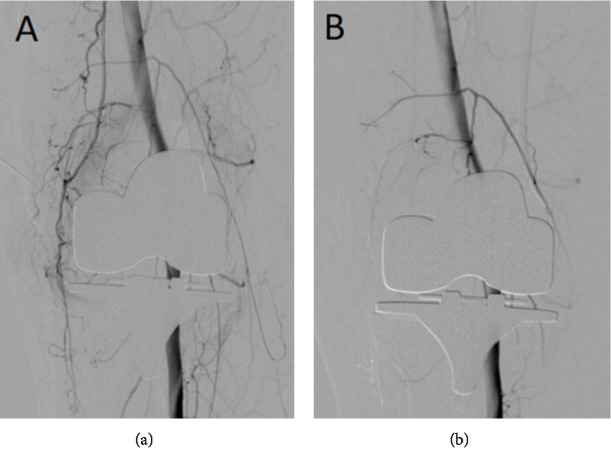
Before and after embolization images. (a) Preembolization image shows an artery “tumor blush” appearance in the lateral and medial genicular arteries, and it is bigger in the medial genicular artery. (b) Postembolization image shows blood flow stasis in the target area and active blood flow in the femoral, popliteal, peroneal, and the anterior and posterior tibial artery.

**Figure 5:**
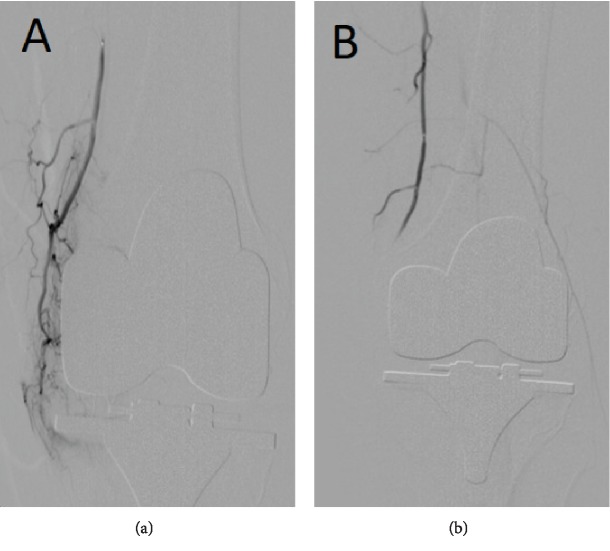
Selective supramedial genicular artery angiography: (a) before embolization and (b) after embolization.

**Figure 6:**
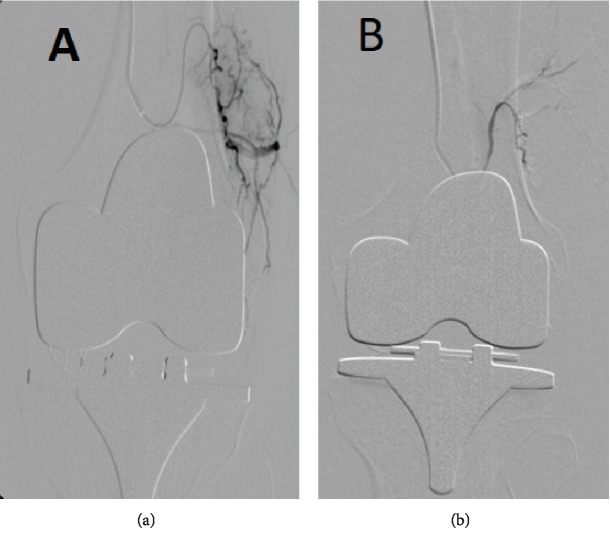
Selective supralateral genicular artery angiography: (a) before embolization and (b) after embolization.

Three weeks after arthroscopy, the patient resumed to have pain and knee effusion. The results of the biopsies were normal synovial tissue. So, angiography was performed (Figure 4), and the patient was readmitted. Angiography shows hyperemic blush in the medial and lateral genicular arteries associated with arterio-arterial anastomoses, being the hyperemic blush greater in the medial side (Figures 5 and 6). The superior medial genicular artery and the superior lateral genicular artery were selectively cannulated with Progreat® 2.7 and Echelon® and were embolized with embospheres of 400-600 *μ*m. Angiography post embolization shows stasis of blood flow in the genicular artery and active blood flow in the femoral, popliteal, peroneal, and the anterior and posterior tibial artery (Figure 4).

**Figure 7:**
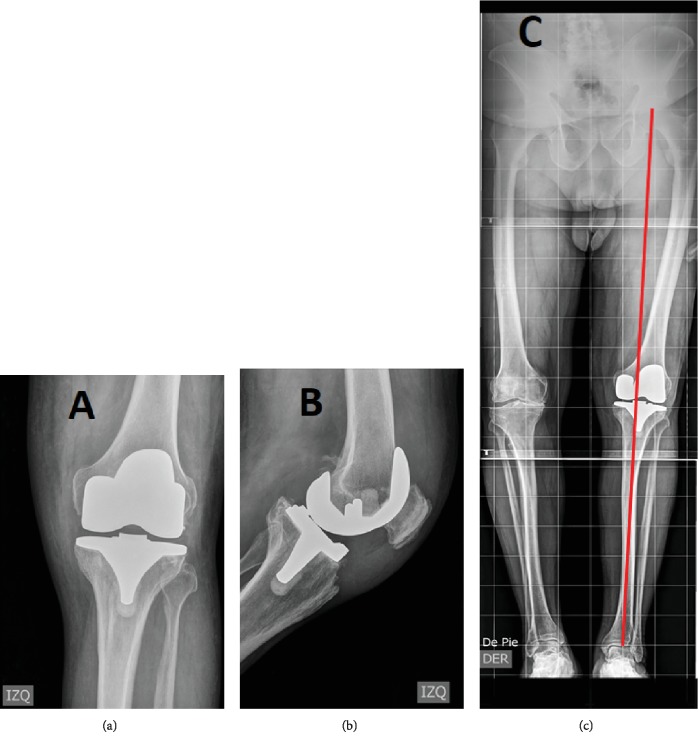
Left knee radiograph two years after embolization: (a) anterior-posterior left knee view, (b) lateral left knee view, and (c) lower anteroposterior limb view with a red line showing the mechanical axis.

Following angiography, the patient did not complain of any pain and was able to resume work. After two years of follow-up, the patient has no complaint of pain or hemarthrosis; he achieved a full extension and 105° of flexion. Radiographs at two-year follow-up are shown in Figure 7. At the two-year follow-up, the patient-reported outcome was 92 in the Forgotten joint score and 0 points in Womac pain, one point in Womac stiffness, and three points in Womac functional score.

## 3. Discussion

Vascular complications (VCs) after knee surgery are rare. Nevertheless, VCs increase the 90-day mortality, risk of infection, and hospital stay, compromising the clinical outcome and the prosthesis survival [5].

Knowing the vascular anatomy is crucial to avoid vascular complications. Three types of popliteal anatomy were described by Tomaszewski et al. according to the level where the popliteal artery divides: type I if it was proximal to the knee joint, type II if it was at the knee joint, or type III if it was below the knee. Type I was the most common reaching an incidence of about 95% [6].

Planning is crucial to avoid VCs. The most critical zone during knee arthroplasty is in the posterior side of the lateral tibial plateau, which if represented in a “clock face” the danger zone is between the 11 and 15 hours [7]. The surgeon must be familiar with the variations of the popliteal trunk division, being type II at higher risk of VC during knee replacement [6].

**Figure 8:**
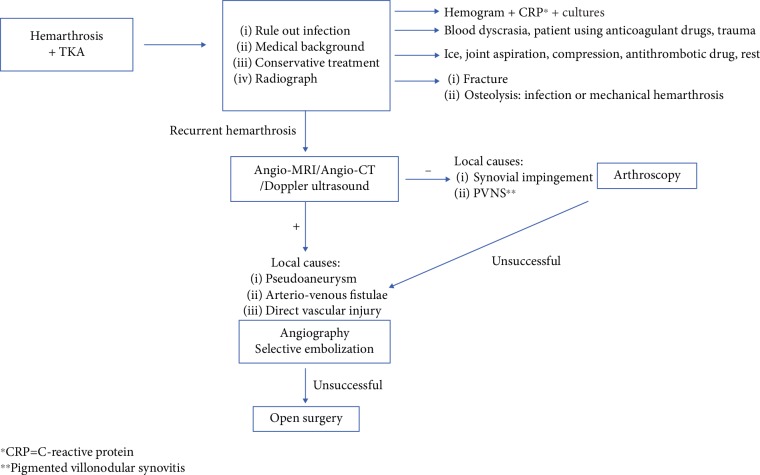
Algorithm proposed for the study and management of hemarthrosis after total knee arthroplasty. ^∗^CRP = C-reactive protein. ^∗∗^Pigmented villonodular synovitis.

Hemarthrosis after knee arthroplasty is a challenging differential diagnosis for the surgeon, the algorithm proposed in this study is summarized in Figure 8. Most patients complain of pain and knee effusion without a history of trauma, so infection should always be ruled out. While performing arthrocentesis, hemarthrosis will be observed. A complete review of the medical history should be performed, with particular emphasis on blood dyscrasia and current or recent use of anticoagulant drugs. A radiograph is always useful to rule out fractures in pathological bone or osteolysis.

Conservative treatment has been reported to have a success rate of 83%, which involves up to three joint aspirations, rest, ice, compression, and antithrombotic drugs [3]. Given the high percentage of success reported, we recommend conservative treatment as initial management; however, physicians must be cautious with the number and frequency of arthrocentesis.

In this case, conservative treatment failed. Given that the time of presentation was seven months after surgery, the most common cause is pseudoaneurysm [4]. Angio-CT elucidates the regional vascular anatomy and can identify active bleeding, being less invasive than to perform angiography. Angio-MRI has the advantage that in addition to the information on the state of the vascular anatomy, it is a radiation-free test and capable of delivering information about the soft tissue around the knee, specifically the synovial tissue. Magnetic resonance angiography (Angio-MRI) considerations include cost and careful image acquisition to minimize the artifact produced by the prosthesis [8]. Literature supports the use of Doppler ultrasound to identify arterial-arterial or arteriovenous fistulae after TKA; however, it should be taken into consideration that it is an operator-dependent exam [9, 10]. To our knowledge, no study has compared the ability to discriminate between Angio-CT, Angio-MRI, or Doppler ultrasound. The decision of what imaging test should be taken is based on the availability, costs, and capacity of the health center; nevertheless, Angio-MRI seems to have an advantage. If a vascular lesion is identified, a selective embolization by angiography should be considered. If any vascular pathology is found, synovial lesions should be suspected.

In this case, the Angio-CT was negative for vascular lesions, so a synovial impingement was suspected. Worland and Jessup [11] reported a case series of synovial impingement; they describe a median presentation of 20 months after surgery with a range between 1 and 30 months. Two of their seven cases had arteriography with no findings, and in all cases, proliferative synovitis was found in histology. Arthroscopy was performed, but no evidence of active bleeding or macroscopic alteration of the synovial tissue was found. Also, biopsies show normal synovial tissue.

As the patient continued with recurrent hemarthrosis, an angiography and a selective embolization were performed. Weidner et al.[12] reported a case of series with successful results in 12/13 cases (92%) of recurrent hemarthrosis after knee arthroplasty; the only failure was a case of misdiagnosed periprosthetic infection. Guevara et al. [13] report a series of cases of 100% success in 10 cases of recurrent hemarthrosis after knee surgery, four of them required a second embolization. Also, Kolber et al. [14] performed a systematic review of outcomes after embolization; they found 91 cases with a recurrence of 10%, two cases of significant complications (periprosthetic infection) and six cases of minor complications including four transient skin ischemia, one persistent pain, and one skin ulceration.

## 4. Conclusions

Recurrent hemarthrosis after knee replacement is uncommon. Success rate after conservative treatment has been reported to be above 80%. In case of recurrence, local complications must be ruled out. Angio-MRI, Angio-CT, and Doppler ultrasound have been proposed to conduct the diagnosis. Evidence for treatment is limited to a series of cases, and in the last decade, it favors the use of selective embolization as it is a successful and safe treatment.
